# Right atrial myxoma as the first manifestation of granulomatosis with polyangiitis, and a possible association with vascular endothelial growth factor (VEGF) and interleukin 6 (IL-6): a case report and review of the literature

**DOI:** 10.1186/s40001-022-00632-z

**Published:** 2022-01-11

**Authors:** Joel Corin, Amanda Carlsson, Björn Peters

**Affiliations:** 1 Department of Nephrology, Skaraborg Hospital, Skövde, Sweden; 2 Department of Molecular and Clinical Medicine, Institute of Medicine, The Sahlgrenska Academy at University of Gothenburg, 41 345 Gothenburg, Sweden

**Keywords:** Case report, Vasculitis, Myxoma, VEGF, IL-6

## Abstract

**Background:**

Granulomatosis with polyangiitis and myxomas are rare conditions previously described to co-exist. Cardiac masses are often presumed to be myxomas rather than lesions of granulomatosis with polyangiitis.

**Case presentation:**

We present a review of the symptoms for the two diagnoses along with the first verified case.

**Conclusions:**

Two possible risk factors for developing myxomas (VEGF and IL-6) are explored and discussed.

## Background

Granulomatosis with polyangiitis (GPA), previously known as Wegener’s granulomatosis, was first described in 1936. GPA is a small- to medium-sized vasculitis histopathological characterised with granulomas [1]. It most commonly affects the respiratory tract and kidneys. Therefore, the first symptoms can include glomerulonephritis, epistaxis, shortness of breath, and haemoptysis. Although rare, the heart is also affected in GPA. A recent large retrospective cohort of 535 patients concluded that 3.3% of patients with GPA had manifestations of the heart [2]. However, numbers vary greatly and in other studies were reported to be over 50% in both new onset patients and for relapsing patients [3, 4]. The pericardium and myocardium are the areas more often affected [2, 4, 5].

Myxomas are primary heart tumours that mainly appear in the left atrial myocardium [1]. Although they are benign, due to the mass effect, they can cause symptoms of heart failure and valvular regurgitation [6]. Severe complication includes embolic clots that result in peripheral ischaemia or stroke [6]. Surgery is often curative if done radically. Possible links between GPA and myxomas were previously documented. A case report from 2014 described a 76-year-old woman with a left atrial wall mass that was discovered with transthoracic electrocardiogram and transoesophageal echocardiogram; the mass shrank spontaneously. The authors initially presumed this cardiac mass to be a myxoma; however, no histopathological examination was documented, and it was later considered to be a lesion of GPA [7]. Similar cases have been described by other authors [8]. In addition, one article presented histopathological verification of a GPA lesion in a patient that was first believed to be an atrial myxoma [9]. However, to our knowledge, no case report has described a patient with GPA and a confirmed myxoma.

## Case presentation

**Fig. 1:**
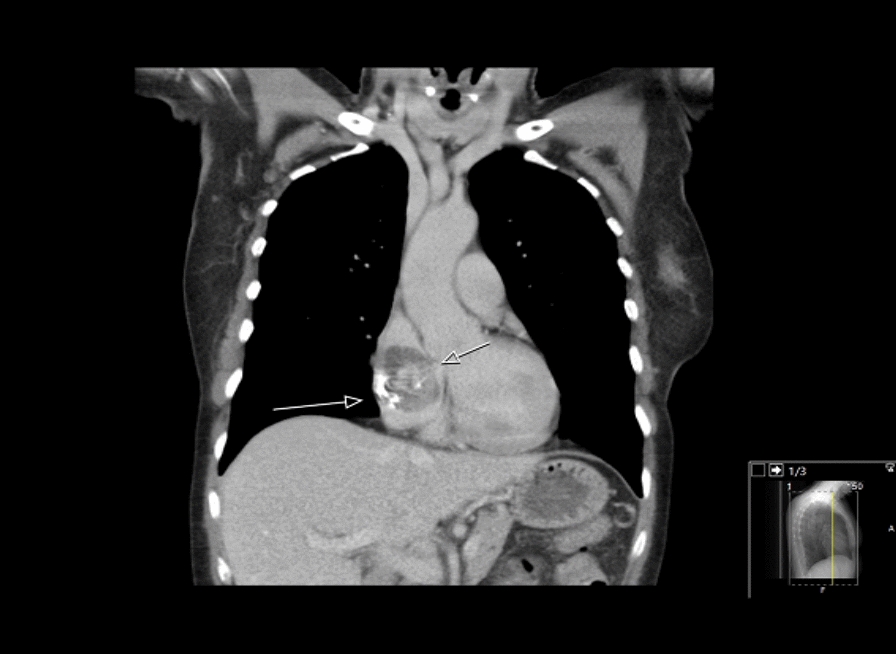
CT-scan of the myxoma in the right atrium

A 58-year-old female with no previously medical history had experienced symptoms for 2 years of recurring sinusitis with periorbital pain, nasal congestion, and fever. Occasionally, blood-strained nasal congestion or nosebleed occurred, and she was diagnosed with chronic maxillary sinusitis. Her symptoms developed to include periodic fatigue, shortness of breath during exercise, nagging cough, ear congestion, and general stiffness without joint pain. Her lab values displayed microcytic anaemia with haemoglobin of 110 g/L (reference 117–153 g/L), mean corpuscular volume (MCV) of 81 femtoliters (reference 82–98 femtoliters) along with signs of inflammation with a C-reactive protein (CRP) of 15 mg/L (reference < 5 mg/L), and erythrocyte sedimentation rate (ESR) of 123 mm/h. Serum creatinine was 52 µmol/L (reference 35–85 µmol/L). The patient with the symptoms and inflammatory parameters was referred from the primary care unit to a diagnostic centre. Furthermore, she underwent a gastroscopy with no source of gastritis or ulcer as a part of an anaemia investigation. The urine protein dipstick test detected no evidence of proteinuria or haematuria. No free light chains and monoclonal immunoglobin were detected in either the blood or urine. Further serological testing was performed, and the outcome was a positive proteinase 3 antineutrophil cytoplasmic antibodies (PR3-ANCA) at the level of 116 IU/mL (reference < 5 IU/mL). The patient was referred to the department of rheumatology. The consideration made was granulomatosis with polyangiitis (GPA) with engagement of the upper respiratory tract with inflammatory activity in lab findings of PR3-ANCA. Induction therapy with 40 mg of Prednisolone per day and 20 mg of Methotrexate once a week was initiated. Prednisolone was then de-escalated biweekly first with 5 mg until a dose of 20 mg was reached. The dose reduction was later lowered by 2.5 mg at the same rate. A nasal mucosal biopsy was performed and revealed pronounced mucosal inflammation. A computer tomography scan revealed a normal appearance of the lungs and a mass of 47 mm in the right atrium (Fig. 1). The patient underwent echocardiography and transoesophageal echocardiogram which revealed a suspicion of cardiac myxoma in the right atrium. A coronary angiogram was performed and showed no evidence of coronary stenosis.

A few months later, the patient underwent a sternotomy at the regional University Hospital for extirpation of the mass in the right atrium. The surgery went without complications. The pathological analysis of the atrial mass displayed no atypia, scattered cells in a faint myxoid stroma and ring-shaped structures around vessels, which confirmed the diagnosis of atrial myxoma. Follow-up echocardiography showed a normal left ventricle ejection fraction > 55%, a mitral valve prolapse with no significant leakage, and a slightly thickened atrial septal. In other words, a normal echocardiogram. One year later, the clinical follow-up showed no relapse, and a new echocardiogram was planned for 3 years. The patient had prompt response to the anti-inflammatory treatment, and both PR3-ANCA and ESR decreased rapidly, to PR3-ANCA of 26 IU/mL and ESR of 14 mm/h. After the myxoma extirpation, the immunosuppressive treatment included Prednisolone with a maintenance dose of 10 mg and Methotrexate at a fixed dose of 20 mg weekly.

**Fig. 2:**
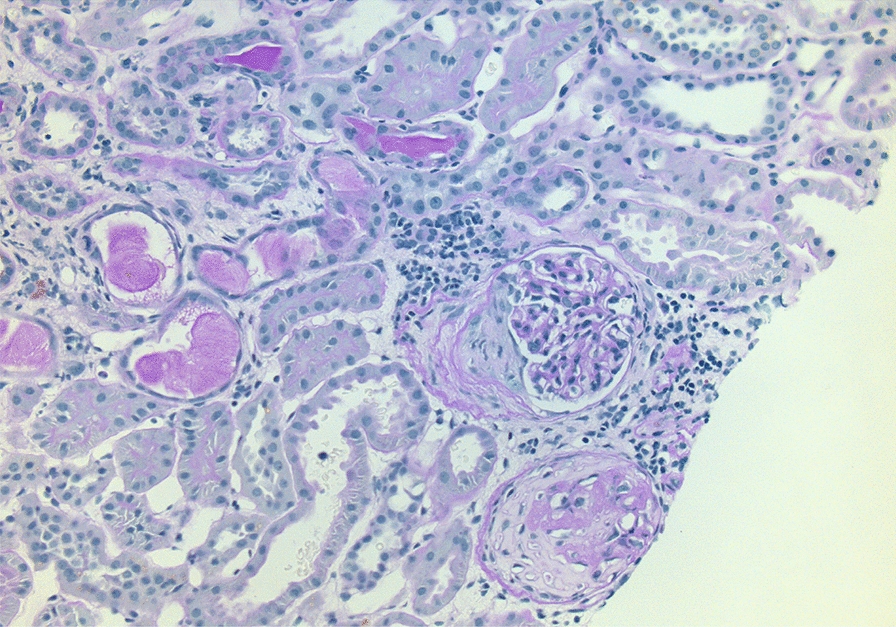
Histopathological findings of kidney biopsy

A year later, the PR3-ANCA and ESR increased again to PR3-ANCA of 112 IU/mL and ESR of 41 mm/h. Serum creatinine was 59 µmol/L. The urine dipstick test detected 2 + proteinuria. Examination of the urine sediment displayed hyaline casts and the presence of 3–5 red blood cells and 5–10 leukocytes per field of view. A kidney ultrasound was performed and showed ordinary kidney size, ordinary parenchymal thickness, and increased echogenicity. On the right kidney, a few parapelvic cysts were detected. There were no signs of hydronephrosis. The patient was admitted for kidney biopsy that identified 27 glomeruli, of which one had the morphology of a crescent and one was completely sclerosed; tubular atrophy was between 10 and 15% (Fig. 2). The biopsy further supported the diagnosis of GPA, now with renal involvement. The current symptoms of ear congestion on the right side and occasional nosebleed were mild compared with the initial onset. A high-resolution computed tomography of the lungs revealed no engagement of the lungs. A new computer tomography of the sinus revealed progression of bone destruction in the maxillary sinuses. Altogether, this was interpreted as a relapse of GPA with multi-organ involvement. An Iohexol clearance was not performed; however, the estimated glomerular filtration rate was 89 ml/min/1.73 m^2^ with serum creatinine of 59 µmol/L as calculated with the Lund–Malmo formula adjusted for age, sex, height, and weight. The dose of Prednisolone was increased to 60 mg followed by de-escalation to 5 mg as a maintenance dose. Additionally, the treatment was intensified with 1000 mg of Rituximab with a second dose after 2 weeks.

Two years after the diagnosis, the patient was treated with 5 mg Prednisolone daily and 20 mg Methotrexate weekly. The patient obtained a second treatment of Rituximab, approximately 1 year after the first treatment. At the last follow-up, GPA was in remission, and there were no new symptoms. The estimated GFR was 65 ml/min/1.73 m^2^ with a serum creatinine of 81 µmol/L and the urine protein dipstick test was negative for both proteins and erythrocytes.

During remission, we tested the serum levels of vascular endothelial growth factor, and it was 131 ng/L (reference 62–707 ng/L). Serum analyses of Interleukin-6 (IL-6) were also performed, before and after stimulation with purified protein derivatives (PPD) and Candida. The patient had lower basal production of IL-6 and less response after stimulation compared with healthy blood donors.

## Discussion and conclusions

Clinical manifestations of the heart in GPA are uncommon. Previous literature suggests that patients with GPA that present with cardiac masses are often inflammatory lesions of GPA and not myxomas [4, 7–9]. However, there might be a common denominator between the two conditions.

Vascular endothelial growth factor (VEGF) is a known angiogenic protein that stimulates the development of new blood vessels. In a study of 46 participants, serum levels of VEGF were considerably elevated in 91.3% of patients with GPA when compared to healthy controls [10]. Moreover, two histopathological studies showed that myxomas often express VEGF that contributes to tumour growth [11, 12]. However, the development of tumour cells is a complex heterogeneous process of genomic instability through activation of oncogenes and suppression of tumour suppression genes. VEGF may most likely not induce oncogenesis; however, the abundance of VEGF in patients with GPA might lower the threshold to develop cancer.

Interleukin-6 (IL-6) is a proinflammatory cytokine that is clinically used, for example, in neonatal sepsis [13]. IL-6 was reported to be elevated in 47 patients with GPA compared to healthy controls, with ANCA-positive patients having even higher IL-6 levels [14]. Additionally, as with VEGF, IL-6 was confirmed to be produced by myxomas and was correlated to tumour size [15–17]. The molecular process for cancerogenic development in myxomas remains unclear, despite some genetic syndromes such as the Carney complex being autosomal dominant for occurrence of myxoma [17, 18].

Repeated echocardiographic screening for GPA manifestations of the heart has been considered and suggested by some authors [3, 7, 19]. Conversely, a study of 89 patients with ANCA-associated vasculitis presented no difference in echocardiographic features when compared to healthy controls [20].

The myxoma in our patient was accidentally found and was removed at a relatively small size of 47 mm. Unfortunately, we have no information on the growth rate and duration of the myxoma, or on the serum levels of VEGF and IL-6 before surgery. The measurement of VEGF and IL-6 was not performed routinely in patients with GPA and myxoma in our clinic. After performing an intensive literature research, we came out with thinking about the measurement of these factors. In future cases with GPA and myxoma, we intend to take these factors routinely already at the time of diagnosis. The patient had normal VEGF levels and low IL-6 levels; we interpret this as GPA is in a clinical remission state and as an effect of the anti-inflammatory treatment.

To our knowledge, this case report is the first to confirm GPA and a verified myxoma. GPA and atrial myxomas could share the same risk factors in proinflammatory cytokines.

## Data Availability

The raw data used in the current study are restricted to protect participant privacy, as required by data protection acts in Sweden. Data can be made accessible by request for researchers after permission from the Swedish Ethics Review Authority.

## Abbreviations

CRP: C-reactive protein
ESR: Erythrocyte sedimentation rate
GFR: Glomerular filtration rate
GPA: Granulomatosis with polyangiitis
IL-6: Interleukin-6
MCV: Mean corpuscular volume
PPD: Purified protein derivatives
PR3-ANCA: Proteinase 3 antineutrophil cytoplasmic antibodies
VEGF: Vascular endothelial growth factor
